# Periodontal Health and Total Antioxidant Capacity for Jordanian Smokers at Dental Teaching Clinic

**DOI:** 10.1155/ijod/3075190

**Published:** 2025-07-21

**Authors:** Noor Al Mortadi, Khaled Al Qudah, Sabha Alshatrat, Karem H. Alzoubi, Rania Mahafdeh

**Affiliations:** ^1^Department of Applied Dental Sciences, Faculty of Applied Medical Sciences, Jordan University of Science and Technology, P.O. Box 3030, Irbid 22110, Jordan; ^2^Department of Clinical Veterinary Medical Sciences, Faculty of Veterinary Medicine, Jordan University of Science and Technology, P.O. Box 3030, Irbid 22110, Jordan; ^3^Department of Clinical Pharmacy, Faculty of Pharmacy, Jordan University of Science and Technology, P.O. Box 3030, Irbid 22110, Jordan; ^4^Department of Clinical Pharmacy, Faculty of Pharmacy, Jadara University, P.O. Box 733, Irbid 21110, Jordan

**Keywords:** antioxidant enzymes, catalase, glutathione peroxidase, periodontitis, smoking oxidative stress, superoxide dismutase

## Abstract

**Background:** Smoking exacerbates oxidative stress and inflammation, both of which contribute to periodontitis. However, few studies have simultaneously assessed/systemic antioxidant capacity, organ function, and hematological indices in smokers with periodontitis. This study aimed to examine antioxidant enzymes, hepatic and renal function, and hematological profiles, offering a comprehensive perspective on smoking-related periodontal damage.

**Methods:** A total of 47 participants were divided into three groups: smokers with periodontitis (*n* = 18), nonsmokers with periodontitis (*n* = 17), and healthy nonsmoking controls without periodontitis (*n* = 12). Salivary and blood samples were collected, and oxidative stress biomarkers, including thiobarbituric acid reactive substances (TBARS) and antioxidant enzymes, such as glutathione (GSH), GSH peroxidase (GPX), superoxide dismutase (SOD), and total antioxidant activity (TAOA), were measured. Additionally, liver function (ALT, AST, GGT, Total bilirubin), kidney function (BUN, creatinine), and hematological parameters were assessed and compared among the three groups.

**Results:** Smokers with periodontitis illustrate significant elevation in TBARS levels in both serum and saliva (*p*  < 0.05), indicating increased oxidative stress compared to nonsmokers and controls. Serum antioxidant enzyme levels (GSH, GPX, SOD, and TAOA) were significantly decreased in the smokers with periodontitis groups (*p*  < 0.05). ALT and AST levels were significantly elevated in the smoking and periodontitis group (44.45 ± 19.64 and 28.27 ± 7.77 U/L), respectively, suggesting significant liver impairment among smokers (*p*  < 0.05). Kidney function was also affected; the BUN and creatinine levels were noticeably higher in smokers compared to the healthy controls and nonsmokers (*p*  < 0.05). Hematological findings exhibit a significant increase in WBCs among smokers-periodontitis patients, indicating an increase in systemic inflammation.

**Conclusion:** The imbalance between oxidative stress and antioxidant capacity may play a role in the pathogenesis of periodontal disease. Smoking is highly linked to oxidative stress-antioxidant (redox) imbalance, which exacerbates the impact of periodontitis. This leads to significant oxidative damage, noticeable liver and kidney dysfunction, and elevated systemic inflammation.

## 1. Introduction

Smoking is a significant public health threat, resulting in increased morbidity and mortality. Worldwide, around 1.3 billion people aged 15 years and above are current smokers, 80% of whom live in low- and middle-income countries [1]. In Jordan, the epidemic of smoking is growing significantly and is considered an urgent public health issue. The estimated prevalence of smoking among adult males (aged 15 years and over) is 70.2%, which is the highest in the MENA region and the second-highest rate globally, after Indonesia. Smoking has a detrimental effect on the occurrence and development of periodontitis [2]. It is considered the second vital modifiable risk factor for periodontal diseases and a causative factor for chronic periodontitis [3, 4]. Periodontitis is a chronic, destructive, inflammatory condition affecting the supporting structures of the teeth, and as such, it is listed as a global burden of chronic diseases [5].

Periodontitis, in its definitive stage, can lead to tooth loss [2]. In Jordan, periodontitis is the leading cause of tooth loss [6]. In meta-analysis study, done by [7] = who reported that the rate of bone loss for smokers is approximately four times that of nonsmokers Periodontal diseases affect about 10%–15% of the world's population, representing the most significant cause of tooth loss In 2021, over 1 billion people were affected by severe periodontitis [8].

A significant relationship exists between periodontitis, antioxidant status, and oxidative stress [9]. Oxidative stress results from an imbalance between reactive oxygen species (ROS) production and antioxidant capacity [10]. If the balance between ROS and antioxidant defense is disturbed in favor of ROS, oxidative stress may occur and may promote the development of periodontitis and dental caries [11].

The antioxidant defense system consists of enzymes, such as catalase, superoxide dismutase (SOD), and glutathione peroxidase (GPX), which decrease the concentrations of oxidants in cells and tissues [12]. Antioxidants neutralize free radicals, ROS, and reactive nitrogen species (RNS), preventing oxidative stress and damage. Interestingly, patients with periodontitis and bad oral health are susceptible to the imbalance between the antioxidant defense and reactive radical generation [13]. This study aims to evaluate the effect of smoking on oxidative stress, antioxidant enzyme levels, and systemic inflammation in patients with periodontitis. Particularly, the study aims to compare the levels of key biomarkers, such as thiobarbituric acid reactive substances (TBARS), glutathione (GSH), SOD, total antioxidant activity (TAOA), and GPX in serum and saliva between smokers and nonsmokers with periodontitis and healthy controls. Additionally, the study seeks to investigate the effects of periodontitis and smoking on liver and kidney function parameters to understand the broader systemic implications of these conditions.

## 2. Materials and Methods

### 2.1. Selection of Patients and Controls

A cross-sectional study was conducted at Jordan University of Science and Technology (JUST) postgraduate dental clinics in Irbid, Jordan, between January and June 2017. The JUST Institutional Review Board (IRB) Committee approved the study. Written informed consent was obtained from all subjects.

The classical parameters for diagnosing periodontitis are clinical parameters, such as probing depths of the gingival crevice, bleeding on probing, and clinical attachment levels previously described by [14]. Participants were diagnosed with periodontitis based on the globally used protocol, depending on specific clinical features, such as pocket depth and attachment loss.

Moderate to severe periodontitis was established using the following clinical criteria: presence of chronic inflammation (pain, redness, heat, swelling), diagnosed by bleeding on probing, at least 5 or 6 sites with probing (pocket) depth ≥ 5 mm, and attachment loss ≥ 3 mm using a probe. The recordings taken during periodontal probing are recorded onto a chart. Six measurements are taken for each tooth, three on the facial side and three on the lingual side. However, a pocket can be anywhere from 1 to 3 mm deep in a healthy mouth. Thus, a control group of 10 individuals with no history of smoking or periodontitis was selected. The participants were recruited voluntarily as they were admitted for their dental appointments.

Patients (*n* = 30) and corresponding healthy controls (*n* = 10) were recruited from JUST postgraduate dental clinics in Irbid, Jordan, using a convenience sampling approach. They were at least 18 years old, in good general physical and mental health, and had a minimum of six teeth in their mouth. Participants with any systemic disease or receiving any therapeutic regimen during the past 6 months were excluded from the study. Participants were subdivided into three groups: Group 1 (healthy control) consisted of 12 healthy people with no sign of periodontitis or smoking. The control subjects were matched to the patient group in terms of sex, age, and socioeconomic status. Group 2 consisted of 18 smokers with chronic periodontitis (smokers & periodontitis who claimed to have smoked at least 10 cigarets per day for the past 5 years at a minimum). Group 3 consisted of 17 nonsmokers who had never smoked but had periodontitis diagnosed (nonsmokers and periodontitis). Although 47 participants were initially enrolled (*n* = 12 controls, *n* = 18 smokers with periodontitis, and *n* = 17 nonsmokers with periodontitis), the number of valid samples analyzed varies slightly across biomarkers due to occasional sample loss or insufficient volume. Exact sample sizes for each group are specified in the corresponding tables.

### 2.2. Data Collection

A questionnaire was developed and divided into three parts. The first part aims to identify the demographic information about participants, including name, age, and gender. The second part reported essential questions, such as clinical presentation (healthy or periodontitis), current and previous medication, and whether the patient is a smoker. For smokers, years of smoking and the number of cigarets/day were recorded. Participants classified as smokers reported daily consumption of at least 10 cigarets for a minimum of five consecutive years. While this threshold ensured chronic exposure, detailed quantification of pack years and smoking intensity was not recorded, which may limit dose–response interpretation. In addition, the reason and frequency for dentist visits, frequency of brushing and mouthwash, frequency of replacing the toothbrush, flossing, type of toothbrush (soft or hard), and the duration of brushing were also recorded. The third part is the clinical presentation, which includes the clinical symptoms, such as pocket depth and loss of attachment.

### 2.3. Saliva Samples Preparation

All participants were instructed to rinse their mouths with water before saliva collection. About 3–5 mL of saliva was collected in Salivette (Sarstedt, Germany) tubes, providing a good method for collecting hygienic saliva. The Salivette contains a plain cotton swab (cotton swab without preparation) to collect unstimulated saliva. The cotton swap was placed under the tongue behind the front teeth for 5 min to collect the saliva according to the manufacturer's instructions. The collected cotton swab was placed in Salivette tubes and centrifuged at 2000 rpm for 5 min at room temperature. The supernatant fraction was stored at −80°C until analysis. Begum et al. [12] used saliva to determine the status of antioxidants.

### 2.4. Blood Samples Preparation

Blood samples were collected aseptically from the median cubital vein using disposable needles. Blood was drawn and transferred into two sealed vacutainer tubes: the first tube containing acid citrate dextrose anticoagulant for the measurements of GSH peroxidase (GSHPx), SOD, and reduced GSH; and the second tube containing lithium heparin anticoagulant for the measurements of TBARS. All samples were placed in an ice box after collection and transferred to the laboratory without delay for analysis to avoid in vitro oxidation. Plasma was immediately separated by centrifugation at 3000 g for 10 min at 4°C, and samples were transferred into vials and stored in a deep freezer at −20°C until analysis of oxidants and antioxidants.

### 2.5. Measurements of Oxidants and Antioxidant Levels

Serum and salivary levels of TBARs and antioxidants, including TAOA, GSH, and enzymatic activities of GSH-Px and SOD, were measured and compared between the three groups. The assay kits for SOD, GSHpx, and GSH reductase were all purchased from Abnova (Abnova GmbH EMBLEM, Heidelberg, Germany). All analyses were performed according to the instructions provided by the manufacturers of the assay kits. All reactions were carried out in duplicate 96-well microplates. Plates were read at a wavelength specified for each kit using a spectrophotometer microplate reader (Biotech, Model Power Wave XS2, Elisa reader). TBARS level was measured using a spectrophotometer (Cecil EC 1021, Model 1000 Series) at 532 nm.

### 2.6. Statistical Analysis

For each biomarker, the data were presented as mean ± SD. Ordinary one-way analysis of variance (ANOVA) was used to find the significance of study parameters by comparing three groups of subjects. Multiple comparisons of each parameter were carried out between the groups using Tukey's Multiple Comparisons Test. Statistical significance is assessed at *p*  < 0.05.

## 3. Results and Discussion

**Figure 1:**
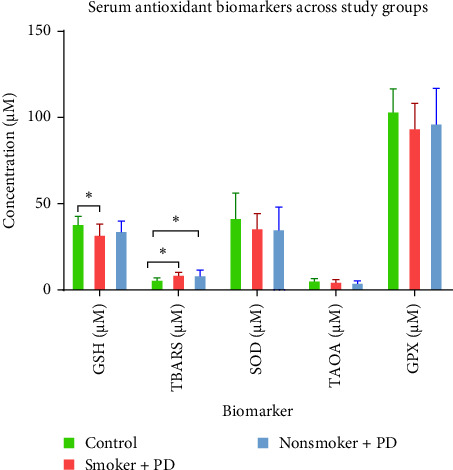
Mean concentrations (± SD) of serum antioxidant and oxidative stress biomarkers: GSH, TBARS, SOD, TAOA, and GPX, in three groups: healthy controls (green, *n* = 10), smokers with periodontitis (red, *n* = 15), and nonsmokers with periodontitis (blue, *n* = 15). Statistical analysis was performed using one-way ANOVA followed by Tukey's post hoc test (*⁣*^*∗*^*p*  < 0.05).

The recruited patients had an average age of years and a male-to-female ratio of 1.35:1. The results showed significant variations in the mean and standard deviation of serum antioxidant enzyme levels between the control and periodontitis groups (Table 1, Figure 1). GSH levels in the serum were significantly decreased in the smoking and periodontitis group (425 ± 89.74 μM) and the nonsmoking and periodontitis group (456 ± 72.32 μM) compared to the healthy controls (596 ± 100 μM) (*p*  < 0.0001). TBARS, a biomarker used as an indicator of lipid peroxidation, was significantly increased in the smoking and periodontitis group (15.12 ± 4.38 μM) compared to both the control (7.7 ± 1.75 μM) and the nonsmoking and periodontitis group (13.4 ± 3.16 μM), exhibited higher oxidative stress in smokers with chronic periodontitis. SOD levels have significantly increased in the control group (0.60 ± 0.05 IR%), in comparison with the smoking and periodontitis group (0.45 ± 0.06 IR%) and nonsmoking and periodontitis group (0.46 ± 0.09 IR%) (*p*  < 0.0001). The total antioxidant capacity, TAOA, was significantly decreased in both smoking and nonsmoking periodontitis (46.8 ± 4.86, 47.14 ± 3.76 nmol/μL), respectively, compared to the control group (52.45 ± 3.88 nmol/μL), (*p*  < 0.05). GPX levels were significantly lower in the smoking and periodontitis group (437 ± 62.44 mU/mL) compared to the control group (517 ± 37.98 mU/mL); however, with the nonsmoking and periodontitis group showing intermediate values (479 ± 38.76 mU/mL) with no significant difference compared to the control or smoking and periodontitis.

Similarly, salivary antioxidant biomarkers of smokers and nonsmokers with chronic periodontitis were slightly lower compared to the healthy controls (Table 2, Figure 1). TBARS were significantly elevated in the saliva of the smoking and periodontitis group (8.18 ± 2.02 μM) and the nonsmoking and periodontitis group (7.96 ± 3.44 μM) compared to the control group. GSH levels also showed a trend toward decreasing in the periodontitis and smoking group (31.4 ± 6.84 μM), though the difference was only borderline significant compared to the controls (*p*=0.0423). Other biomarkers, SOD, TAOA, and GPX, did not show significant differences among the three groups.

The results of hematological parameters for control, smokers, and nonsmokers periodontitis patients (Table 3) showed that HGB levels were significantly increased in the smoking and periodontitis groups (14.69 ± 1.06 g/dL) compared to the control group (13.28 ± 1.02 g/dL) (*p*  < 0.05). WBCs were also significantly elevated in the smoking and periodontitis group (9.63 ± 2.05 × 10^3^/μL), compared with the control (7.13 ± 1.40 × 10^3^/μL), (*p*  < 0.05). However, leukocyte differential count showed minor variations among the groups, with no significant variations among the three groups. HCT% was higher in the smoking and periodontitis group (43.07% ± 2.26%) compared to the control group (40.44% ± 2.94%) and the nonsmoking and periodontitis group (42.72% ± 3.52%). Platelet count showed lower values in the periodontitis groups; the mean value for the nonsmoking group was (270 ± 59.00 × 10^3^/μL) and for the smoking group, it was (286 ± 61.00 × 10^3^/μL), compared to 298 ± 61.00 × 10^3^/μL in the control group. Meanwhile, the values of MCHC and MCV were similar across all groups.

The liver and kidney function parameters for the control group, smoking and periodontitis group, as well as the nonsmoking and periodontitis group, are shown in Table 4. The smoking and periodontitis group exhibited the highest BUN levels (5.00 ± 1.060 mg/dL) compared to the control group, which had the lowest levels (4.08 ± 0.770 mg/dL), and the nonsmoking and periodontitis group (4.71 ± 0.960 mg/dL). The mean albumin values in the smoking and periodontitis group were higher (43.11 ± 2.11 g/L) compared to the control and the nonsmoking and periodontitis group (42.62 ± 3.50 g/L, 43.38 ± 3.00 g/L), respectively. Total bilirubin levels were elevated in the smoking and periodontitis group (14.73 ± 4.24 μmol/L). GGT levels were also increased in the smoking and periodontitis group (28.90 ± 13.09 U/L) compared to the control group (25.40 ± 7.54 U/L) and the nonsmoking group (23.62 ± 8.18 U/L). However, the BUN, total protein, GGT, and albumin levels did not present significant changes among the three groups (*p* > 0.05).

While creatinine levels were significantly higher in the smoking and periodontitis group (79.90 ± 7.32 μmol/L) compared to the control group (49.8 ± 19.07 μmol/L) and the nonsmoking and periodontitis group (55.50 ± 19.44 μmol/L) (*p*  < 0.05), ALT levels were significantly elevated (*p*  < 0.05) in the smoking and periodontitis group (44.45 ± 19.64 U/L), in comparison to the control group (19.90 ± 6.67 U/L) and the nonsmoking group (21.56 ± 10.96 U/L). Similarly, AST levels were significantly higher in the smoking and periodontitis group (28.27 ± 7.77 U/L) relative to the control group (21.00 ± 6.44 U/L) and the nonsmoking group (19.25 ± 5.75 U/L) (*p*  < 0.05).

Periodontitis is a complex inflammatory disease that affects the supporting structures of the dentition. Several factors, the most important of which is smoking, can alter the severity of periodontitis [15]. Smoking is one of the prevalent unhealthy habits among individuals in developing countries [16]. Smoking has been clearly shown to be a risk factor for periodontitis [15]. The etiology of smoking-induced periodontal disorders is linked to several physiological and molecular pathways, including immunosuppression and increased inflammatory responses [16, 17]. Our study evaluates the various antioxidant enzyme levels (GSH, SOD, TAOA, and GPX), as well as oxidative stress markers (TBARS) in both serum and saliva; hematological parameters and liver and kidney function were evaluated with periodontitis, either smokers or nonsmokers, compared to the healthy controls.

Tissue destruction by oxidative stress can be measured by measuring the by-product of lipid peroxidation, such as TBARS [17]. Our results showed that the mean levels of TBARS were highest in smokers with chronic periodontitis compared to healthy controls and nonsmoking chronic periodontitis subjects. These findings align with previously reported studies [15, 17–20]. Lipid peroxidation proceeds as one of the most significant reactions of free radicals [15, 17]. Increased lipid peroxidation levels have previously been reported in inflammatory periodontal tissue, which may contribute to the degenerative processes of periodontitis [15, 21]. Furthermore, the mean levels of the antioxidant biomarkers (GSH, SOD, TAOA, and GPX) in saliva and serum of smoking and periodontitis patients were decreased compared to healthy controls and nonsmoking and periodontitis. Our findings exhibited that the antioxidant capacity in saliva and serum was inversely associated with smoking and chronic periodontitis. These findings are aligned with previously published studies [15, 22–25]. Cigaret smoke contains more than 7000 chemicals, such as nicotine, tar, acrolein, formaldehyde, and heavy metals (e.g., cadmium and lead) [26], which contribute to oxidative damage by generating ROS and decreasing endogenous antioxidants like GSH [27]. Nicotine itself has been shown to enhance the production of inflammatory cytokines, such as TNF-α and IL-6, which increase oxidative damage in periodontal tissues [28]. Tar and polycyclic aromatic hydrocarbons can disrupt mitochondrial function and impair antioxidant enzyme activity. Chronic exposure to these toxins can downregulate the expression of antioxidant genes, leading to a sustained redox imbalance [29]. These mechanisms explain the significantly higher levels of TBARS and reduced antioxidant capacity observed in smokers with periodontitis in our study. Previous studies showed that smoking increases free radicals in periodontal tissues and affects the antioxidant capacity. For example, in a recently published study, Iyer and his colleagues 2023 reported that salivary reduced-GSH and total antioxidants were lower among smokers, and periodontal status was significantly poorer among smokers [23]. Furthermore, Ghazi et al. [30] also revealed the adverse effects of smoking on lipid peroxidation and GSHPx activity in saliva in periodontal disease [30]. Findings support this, suggesting that extracellular GSHPx gene expression is induced by chronic exposure to cigaret smoke [31]. While salivary TBARS levels showed significant differences across groups, other salivary biomarkers, such as GSH, SOD, and GPX did not reach statistical significance. This contrast with the serum findings may be due to several factors: lower concentration of enzymes in saliva, variability in salivary flow, or differences between local and systemic oxidative stress dynamics. Although saliva is a promising diagnostic fluid due to its noninvasive collection, these findings suggest that its utility may be more limited for specific systemic biomarkers unless further standardized and validated. Future studies should explore the correlation between salivary and serum biomarkers to better define the role of saliva in oxidative stress assessment in periodontitis. Additionally, in our research, we did not assess participants' dietary intake, which may have influenced these biochemical outcomes, as the nutritional status plays a crucial role in maintaining redox balance, particularly in the context of inflammatory diseases, such as periodontitis [32, 33]. Previous studies have demonstrated that diets rich in antioxidants, such as vitamin C, vitamin E, polyphenols, and carotenoids, can enhance antioxidant defense systems and reduce periodontal tissue inflammation [32, 34, 35]. This is an important consideration for future research to distinguish the contribution of nutritional factors from disease- or smoking-related oxidative stress.

Further, in our study, the hematological parameters showed that lymphocytes and neutrophils show slight variations between the control and periodontitis groups, which could reflect chronic inflammation. The results also showed significant changes in white blood cell counts among the groups; the significant variations observed between the control and periodontitis groups could reflect chronic inflammation. The elevated WBC count in the smoking and periodontitis group may suggest an enhanced inflammatory response, as smoking is known to increase systemic inflammation. These findings align with previous studies [36–38]. For example, Kanakdande et al. [39] have revealed that cigaret smoking is the primary risk factor in the incidence as well as the progression of periodontal disease; it also impacts white blood cell and red blood cell counts [39]. Nishida and his colleagues previously suggested in 2005 that inflammation might be why smokers exhibit poor periodontal status [40]. According to Erdemir et al. [41], smokers with chronic periodontitis had fewer erythrocytes, a lower value of HGB, and lower hematocrit and iron than nonsmokers with chronic periodontitis [41]. Based on our hematological findings, we suggest that the systemic inflammatory response associated with smoking and periodontitis may be more generalized, rather than involving shifts in specific leukocyte populations. Additionally, the sample size may have been insufficient to detect more precise differences in these subtypes. These findings align with prior reports that WBC count is a sensitive marker of systemic inflammation, even in the absence of changes in leukocyte distribution.

Moreover, the liver and kidney function were inversely affected by smoking and periodontitis. Creatinine levels were significantly increased in the smoking group compared to the healthy control and nonsmokers. This elevation in creatinine levels in the smoking and periodontitis groups suggests a possible early negative impact on kidney function. The smoking groups exhibited a significant elevation in ALT and AST levels. This implies that smoking, combined with periodontitis, could negatively impact liver function. The liver is crucial in detoxifying toxic substances and managing oxidative stress. This elevation in liver enzymes may be a result of oxidative liver damage brought on by smoking and chronic inflammation from periodontitis. Our findings are consistent with other research that shows smoking aggravates liver impairment, especially when combined with long-term inflammatory diseases like periodontitis. This study provides a novel assessment of both salivary and serum biomarkers, including antioxidant enzymes, liver and kidney function tests, and hematological parameters. This provides a comprehensive overview of the systemic impact of smoking and periodontitis, unlike many previous studies that focused on single fluid or limited outcome measures.

However, our study has a number of limitations. First, the sample size was relatively small (*n* = 47), although statistically significant results were observed; the small sample size may affect the generalizability of the findings. Second, there were slight inconsistencies in group sizes across analyses due to the exclusion of some samples with insufficient volume or quality. Third, smoking detailed quantification (e.g., pack years or intensity levels) was not assessed. This restricts the ability to explore potential dose-dependent effects of smoking on oxidative stress and periodontal parameters. Future studies should consider either matching groups or adjusting for these confounders in statistical models. Fifth, the study did not collect data on participants' dietary intake or nutritional status. The absence of nutritional data limits the interpretation of biochemical results. Future studies are needed to make dietary assessments for nutritional intake.

## 4. Conclusions

This study focused on the interaction between smoking and periodontitis and their dual impact on systemic oxidative stress, organ function, and immunological function. Smokers with periodontitis exhibit a more noticeable decrease in antioxidant enzyme activity. These findings highlight the significance of smoking cessation in patients with periodontitis to alleviate systemic inflammation and organ dysfunction. Further research is needed to evaluate the long-term effects of smoking on systemic health and to explore the specific components of cigaret smoke responsible for this relationship and by which mechanisms exert their effects.

## Figures and Tables

**Table 1:** Mean ± standard deviation of serum antioxidant enzymes in three groups (control, periodontitis and smoking, and periodontitis and nonsmoking) with statistical comparison using one-way ANOVA followed by Tukey's Multiple Comparisons Test.

Parameter	Serum biomarkers				
	Mean ± SD				
	Control, *N* = 10	Smoking and periodontitis, *N* = 15	Nonsmoking and periodontitis, *N* = 15	*p*-Value	
GSH (µM)	596 ± 100	425 ± 89.74	456 ± 72.32	<0.0001*⁣*^*∗*^	
TBARS (µM)	7.7 ± 1.75	15.12 ± 4.38	13.4 ± 3.16	<0.0001*⁣*^*∗*^	
SOD (IR%)	0.60 ± 0.05	0.45 ± 0.06	0.46 ± 0.09	<0.0001*⁣*^*∗*^	
TAOA (nmol/µL)	52.45 ± 3.88	46.8 ± 4.86	47.14 ± 3.76	0.0044*⁣*^*∗*^	
GPX (mU/mL)	517 ± 37.98	437 ± 62.44	479 ± 38.76	0.0011*⁣*^*∗*^	

**Multiple comparisons of three groups using Tukey's test**					
	**GSH**	**TBARS**	**SOD**	**TAOA**	**GPX**

Control healthy group vs. periodontitis and smoking	<0.0001*⁣*^*∗*^	<0.0001*⁣*^*∗*^	<0.0001*⁣*^*∗*^	0.0065*⁣*^*∗*^	0.0008*⁣*^*∗*^
Control healthy group vs. periodontitis and nonsmoking	0.0009*⁣*^*∗*^	0.0007*⁣*^*∗*^	<0.0001*⁣*^*∗*^	0.0109*⁣*^*∗*^	0.1523
Periodontitis and smoking vs. periodontitis and nonsmoking	0.5920	0.3656	0.9214	0.9737	0.0612

*Note:* Sample sizes reflect only those with complete data for the corresponding parameter. Some group sizes differ due to excluded or inadequate samples.

*⁣* ^*∗*^ *p*  < 0.05 denotes a significant factor.

**Table 2:** Mean ± standard deviation of salivary antioxidant enzymes in three groups (control, periodontitis and smoking, and periodontitis and nonsmoking) with statistical comparison using one-way ANOVA followed by Tukey's Multiple Comparisons Test.

Parameter	Salivary biomarkers				
	Mean ± SD				
	Control, *N* = 11	Periodontitis and smoking, *N* = 15	Periodontitis andnonsmoking, *N* = 14	*p*-Value	
GSH (µM)	37.63 ± 5.02	31.4 ± 6.84	33.57 ± 6.41	0.0528	
TBARS (µM)	5.20 ± 1.76	8.18 ± 2.02	7.96 ± 3.44	0.0114*⁣*^*∗*^	
SOD (IR%)	41.10 ± 15.10	35.06 ± 9.25	34.5 ± 13.56	0.3746	
TAOA (nmol/µL)	4.87 ± 1.73	4.10 ± 1.87	3.57 ± 1.64	0.2024	
GPX (mU/mL)	102.8 ± 13.93	93.19 ± 14.97	95.85 ± 21.03	0.3663	

**Multiple comparisons of three groups using Tukey's test**					
	**GSH**	**TBARS**	**SOD**	**TAOA**	**GPX**

Control healthy group vs. periodontitis and smoking	0.0423*⁣*^*∗*^	0.0153*⁣*^*∗*^	0.4559	0.5217	0.3436
Control healthy group vs. periodontitis and nonsmoking	0.2523	0.0288*⁣*^*∗*^	0.4038	0.1754	0.5763
Periodontitis and smoking vs. periodontitis and nonsmoking	0.6215	0.9709	0.9921	0.7012	0.9082

*Note:* Sample sizes reflect only those with complete data for the corresponding parameter. Some group sizes differ due to excluded or inadequate samples.

*⁣* ^*∗*^ *p*  < 0.05 denotes a significant factor.

**Table 3:** Mean ± standard deviation of hematological parameters in three groups (control, periodontitis and smoking, and periodontitis and nonsmoking) with statistical comparison using one-way ANOVA followed by Tukey's Multiple Comparisons Test.

Parameter	Salivary biomarkers			
	Mean ± SD			
	Control, *N* = 12	Periodontitis and smoking, *N* = 15	Periodontitis and nonsmoking, *N* = 13	*p*-Value
HCT (%)	40.44 ± 2.94	43.07 ± 2.26	42.72 ± 3.52	0.0594
HGB (g/dL)	13.28 ± 1.02	14.69 ± 1.06	13.90 ± 0.92	0.0033*⁣*^*∗*^
RBC (10^6^/µL)	4.85 ± 0.54	5.21 ± 0.40	4.96 ± 0.41	0.1127
WBC (10^3^/µL)	7.13 ± 1.40	9.63 ± 2.05	8.48 ± 2.23	0.0081*⁣*^*∗*^
PLTS (10^3^/µL)	298 ± 61.00	286 ± 61.00	270 ± 59.00	0.5129
MCH (pg)	27.53 ± 2.02	28.82 ± 2.25	27.79 ± 1.80	0.2265
MCHC (g/dL)	32.83 ± 0.72	33.30 ± 0.70	33.03 ± 0.64	0.2168
MCV (fL)	83.78 ± 4.90	87.04 ± 5.49	84.03 ± 4.12	0.1613
Lymphocytes (10^3^/µL)	29.04 ± 7.82	33.10 ± 9.82	28.45 ± 5.56	0.2597
Monocytes (10^3^/µL)	5.86 ± 1.07	6.56 ± 1.45	7.17 ± 1.74	0.0934
Neutrophils (10^3^/µL)	63.83 ± 7.76	58.37 ± 10.71	62.46 ± 4.86	0.2122
Basophils (10^3^/µL)	0.36 ± 0.12	0.32 ± 0.13	0.34 ± 0.08	0.6602
Eosinophils (10^3^/µL)	1.34 ± 0.83	1.46 ± 0.85	1.56 ± 0.87	0.8124

**Multiple comparisons of three groups using Tukey's test**				
	**Control healthy group vs. periodontitis and smoking**	**Control healthy group vs. periodontitis and nonsmoking**	**Periodontitis and smoking vs. periodontitis and nonsmoking**	

HCT (%)	0.0645	0.1388	0.9464	
HGB (g/dL)	0.0024*⁣*^*∗*^	0.2835	0.1089	
RBC (10^6^/µL)	0.1102	0.8146	0.3173	
WBC (10^3^/µL)	0.0057*⁣*^*∗*^	0.2065	0.2756	
PLTS (10^3^/µL)	0.8653	0.4849	0.7652	
MCH (pg)	0.2463	0.9460	0.3881	
MCHC (g/dL)	0.1951	0.7492	0.5588	
MCV (fL)	0.2126	0.9911	0.2501	
Lymphocytes (10^3^/µL)	0.4024	0.9817	0.2908	
Monocytes (10^3^/µL)	0.4366	0.0762	0.5164	
Neutrophils (10^3^/µL)	0.2196	0.9109	0.4043	
Basophils (10^3^/µL)	0.6346	0.8980	0.8869	
Eosinophils (10^3^/µL)	0.9296	0.7958	0.9484	

*Note:* Sample sizes reflect only those with complete data for the corresponding parameter. Some group sizes differ due to excluded or inadequate samples.

*⁣* ^*∗*^ *p*  < 0.05 denotes a significant factor.

**Table 4:** Mean ± standard deviation of liver and kidney function parameters in three groups (control, periodontitis and smoking, and periodontitis and nonsmoking) with statistical comparison using one-way ANOVA followed by Tukey's Multiple Comparisons Test.

Parameter	Salivary biomarkers			
	Mean ± SD			
	Control, *N* = 10	Periodontitis and smoking, *N* = 11	Periodontitis and nonsmoking, *N* = 11	*p*-Value
BUN (mg/dL)	4.08 ± 0.770	5.00 ± 1.060	4.71 ± 0.960	0.2080
Creatinine (μmol/L)	49.8 ± 19.07	79.90 ± 7.32	55.50 ± 19.44	0.0004*⁣*^*∗*^
Total protein (g/L)	73.66 ± 3.42	70.45 ± 3.68	73.76 ± 3.43	0.0597
Albumin (g/L)	42.62 ± 3.50	43.11 ± 2.11	43.38 ± 3.00	0.8339
Total bilirubin (μmol/L)	12.46 ± 2.50	14.73 ± 4.24	10.93 ± 2.58	0.0329*⁣*^*∗*^
ALT (U/L)	19.90 ± 6.67	44.45 ± 19.64	21.56 ± 10.96	0.0083*⁣*^*∗*^
AST (U/L)	21.00 ± 6.44	28.27 ± 7.77	19.25 ± 5.75	0.0092*⁣*^*∗*^
GGT (U/L)	25.40 ± 7.54	28.90 ± 13.09	23.62 ± 8.18	0.4618

**Multiple comparisons of three groups using Tukey's test**				
	**Control healthy group vs. periodontitis and smoking**	**Control healthy group vs. periodontitis and nonsmoking**	**Periodontitis and smoking vs. periodontitis and nonsmoking**	

BUN (mg/dL)	0.1909	0.4482	0.8329	
Creatinine (μmol/L)	0.0006*⁣*^*∗*^	0.7016	0.0038*⁣*^*∗*^	
Total protein (g/L)	0.1095	0.9977	0.0866	
Albumin (g/L)	0.9214	0.8219	0.9742	
Total bilirubin (μmol/L)	0.2583	0.5314	0.0261*⁣*^*∗*^	
ALT (U/L)	0.0159*⁣*^*∗*^	0.9780	0.0218*⁣*^*∗*^	
AST (U/L)	0.0491*⁣*^*∗*^	0.8231	0.0102*⁣*^*∗*^	
GGT (U/L)	0.7049	0.9127	0.4401	

*Note:* Sample sizes reflect only those with complete data for the corresponding parameter. Some group sizes differ due to excluded or inadequate samples.

*⁣* ^*∗*^ *p*  < 0.05 denotes a significant factor.

## Data Availability

The data that support the findings of this study are available from the corresponding author upon reasonable request.
